# High Improvement in Lactic Acid Productivity by New Alkaliphilic Bacterium Using Repeated Batch Fermentation Integrated with Increased Substrate Concentration

**DOI:** 10.1155/2019/7212870

**Published:** 2019-01-17

**Authors:** Mohamed Ali Abdel-Rahman, Saad El-Din Hassan, Mohamed Salah Azab, Abdullah-Al- Mahin, Mahmoud Ali Gaber

**Affiliations:** ^1^Botany and Microbiology Department, Faculty of Science (Boys), Al-Azhar University, P.N.:11884, Nasr City, Cairo, Egypt; ^2^Microbiology and Industrial Irradiation Division (MIID), Institute of Food and Radiation Biology (IFRB), Atomic Energy Research Establishment (AERE), Ganakbari, Savar, Dhaka-1349, Bangladesh

## Abstract

Optically pure lactic acid (LA) is an important chemical platform that has a wide range of industrial and biotechnological applications. Improved parameters for cost effective LA production are of great interest for industrial developments. In the present study, an alkaliphilic lactic acid bacterium, BoM 1-2, was selected among 369 newly obtained bacterial isolates. It was characterized using API 50 CHL kit and identified as* Enterococcus hirae* BoM 1-2 by 16S rRNA gene sequence analysis. Efficient polymer-grade L-lactic acid production was achieved at pH 9.0 and 40°C. In batch fermentation strategy using 20 g L^−1^ glucose, 19.6 g L^−1^ lactic acid was obtained with volumetric productivity of 2.18 g L^−1 ^h^−1^. While using 100 g L^−1^ glucose, 96.0 g L^−1^ lactic acid was obtained with volumetric productivity of 1.07 g L^−1 ^h^−1.^ The highest lactic acid concentration of 180.6 g L^−1^ was achieved in multipulse fed batch strategy with volumetric productivity of 0.65 g L^−1 ^h^−1^. To achieve higher productivity, repeated fermentation processes were applied using the two different strategies. In the first strategy, the lactic acid productivity was increased from 1.97 g L^−1 ^h^−1^ to 4.48 g L^−1 ^h^−1^ when the total of 10 repeated runs were carried out using 60 g L^−1^ glucose, but lactic acid productivity decreased to 2.95 g L^−1 ^h^−1^ using 100 g L^−1^ glucose. In second strategy, repeated fermentation coupled with gradual increase in glucose concentration from 40 to 100 g L^−1^ was conducted for 24 runs. A dramatic increase in LA productivity up to 39.9 g L^−1 ^h^−1^ (18-fold compared to first run) was achieved using 40 g L^−1^ glucose while volumetric productivity ranging between 24.8 and 29.9 g L^−1 ^h^−1^ was achieved using 60–100 g L^−1^ glucose.

## 1. Introduction

Lactic acid (LA) is an industrially important platform chemical that is utilized as pH regulator and preservatives for food and beverages sector and for cosmetics, textiles, pharmaceuticals, and chemical industries. It has received more interest in recent years as a raw material for production of biodegradable plastics (poly-lactic acid, PLA) [1]. Global LA demands were estimated to be 1,947.2 kilo tons in 2018 and are expected to grow annually by 16.2% from 2019 to 2025 as a result of increasing sales of medicines and perfumes in different countries (http://www.grandviewresearch.com/press-release/global- lactic-acid-and-poly-lactic-acid-market).

Lactic acid has two optical isomers [l- and d-lactic acid], which only can be produced via microbial fermentation as chemical synthesis produces racemic mixture [dl-LA]. Biotechnological LA production is mostly driven by wild types or genetically engineered lactic acid bacteria (LAB) that can produce LA with high concentration, yield, and productivity either through homofermentative or heterofermentative pattern based on the fermentation end product [2]. Homofermentative LAB are of interest for commercial scale lactic acid production that produces lactic acid as the major end product without by-products. However, most industrially applicable LAB are mesophilic (optimal temperature ≤ 37°C) and/or neutrophilic (optimal pH 7.0) facilitating contamination risk with unwanted microbes that might decrease fermentation efficiency and affect the end product quality [1]. Therefore, utilization of thermotolerant LAB strains might reduce this risk. Besides, utilization of alkaliphilic strains is advantageous not only for minimizing contamination problems due to their tolerance to high salt levels but also for reducing the neutralizing agents required during LA fermentation [3]. Few alkaliphilic LA producer strains have been reported including* Halolactibacillus halophilus*,* Exiguobacterium sp.*,* Enterococcus spp.*, and* Bacillus *spp, but achieving low LA productivity [4–7].

The efficiency of lactic acid fermentation is mainly dependent on the producer strain and fermentation strategy. Batch fermentation strategy is the simplest process but it suffers from low cell biomass and low LA productivity owing to exhausted nutrients, substrate and/or product inhibition, and elongated fermentation time [8]. Fed batch fermentation strategy might overcome substrate inhibition and results in high LA concentration but also resulted in low LA productivity [9]. On the other hand, repeated fermentation strategy that involves repeated cycles by inoculating a part or all of the free or immobilized cells from a previous run into the next run has reported several advantages in saving labor and seed preparation time and achieving high LA productivity [10]. This fermentation mode is usually conducted using the same substrate concentrations in several runs that might result in increased LA productivity or attained the same productivity as initial run.

This study focused on the challenge to increase LA concentration and LA productivity from high glucose concentration using repeated batch fermentation. Additionally, new isolated alkaliphilic LAB were used in order to avoid the risk of contamination with other microbes that might reduce fermentation efficiency. We also aimed to optimize the LA production by investigating the physical and nutritional requirements as well as fermentation strategies. Therefore, establishment of new strategy for high improvement of LA productivity via investigating the effect of different sugar concentrations coupled with repeated fermentation was conducted for commercial application purposes.

## 2. Materials and Methods

### 2.1. Isolation and Screening of Alkaliphilic LAB

Different soil and water samples as well dairy products were collected and used as isolation sources. One gram or mL was mixed with 100 mL of saline solution (0.85% NaCl), and then 5 mL of the suspensions were added to 100 mL-Erlenmeyer flask containing 50 mL of enrichment MRS medium (MRS) and incubated at 37°C for 48 h. MRS media consisted of 20 g L^−1^ glucose, 10 g L^−1^ peptone, 10 g L^−1^ beef extract, 4 g L^−1^ yeast extract, 5 g L^−1^ sodium acetate, 2 g L^−1^ ammonium citrate, 2 g L^−1^ K_2_HPO_4_, 0.1 g L^−1^ MgSO_4_, 0.5 g L^−1^ MnSO_4_, and 1 mL Tween 80. All chemicals are purchased from Sigma unless otherwise mentioned. The obtained bacterial colonies were grown on MRS agar medium supplemented with 0.4 g L^−1^ bromocresol green to indicate acid production. Positive acid-producing isolates were tested for catalase activity using 3% H_2_O_2_ for primary selection of lactic acid bacteria. All bacterial isolates were kept at −80°C in MRS media containing 15% glycerol. Catalase positive isolates were grown on MRS broth medium without/with supplementation of 50 and 100 g L^−1^ glucose or 10 and 20 g L^−1^ of sodium acetate, separately, and incubated at 37°C for 30 h for quantitative determination of lactic acid.

### 2.2. Characterization and Identification of Isolate BoM1-2

Culture characteristics of the highest LA producer isolate BoM1-2 were determined on MRS agar plates. Gram reaction, cell shape, and arrangements were investigated using Gram stain. Biochemical and physiological characteristics were determined using API 50 CHL test kit (bioMerieux, Marcy l' Étoile, $\overset{´}{F}$rance) for sugar assay, growth at different temperatures, and growth at different concentrations of sodium chloride. Various enzymatic activities were assayed as previously described [11]. Genomic DNA was extracted according to the method of Miller et al. [12]. Bacterial 16S rRNA genes were amplified using the genomic DNA as template and bacterial universal primers of 27 f (5-GAGTTTGATCACTGGCTCAG-3) and 1492 r (5-TACGGCTACCTTGTTACGACTT-3) [13]. The PCR mixture contained 1x PCR buffer, 0.5 mM MgCl_2_, 2.5 U Taq DNA polymerase (QIAGEN, Germantown, MD 20874, USA), 0.25 mM dNTP, 0.5 *μ*M of each primer, and 1 *μ*L of extracted genomic DNA. The PCR was performed in a DNA Engine Thermal Cycler (PTC-200, BIO-RAD, USA) with 94°C for 3 min, followed by 30 cycles of 94°C for 30 s, 55°C for 30 s, and 72°C for 1 min, followed by a final extension performed at 72°C for 10 min. The PCR products were checked for the expected size on 1% agarose gel and purified using GeneJET™ PCR Purification Kit (Thermo K0701). The PCR products were sequenced on GATC Company by use ABI 3730xl DNA sequencer by using forward and reverse primers. The sequences were compared against the GenBank database using the NCBI BLAST research. Multiple sequence alignment was done using ClustalX 1.8 software package (http://www-igbmc.u-strasbg.fr/BioInfo/clustalx/) and a phylogenetic analysis was constructed by the maximum likelihood method based on the Kimura two-parameter model using MEGA (Version 6.1) software, with confidence tested by bootstrap analysis (1000 repeats).

### 2.3. Optimization of Fermentation Conditions

To determine the optimal pH for LA production by strain* E. hirae* BoM1-2, batch fermentation processes were conducted at 37°C in the 250 mL-Erlenmeyer flasks containing 100 mL working volume of MRS medium containing 20 g L^−1^ glucose. The flasks were inoculated at 10% from preculture incubated at 37°C for 30 h. The pH was adjusted at 5.0, 6.0, 7.0, 8.0, 8.5, 9.0, 10.0, and 11.0 using intermitted pH control with 10 N NaOH. Samples were taken periodically every 3–6 h to analyze cell growth (OD_600nm_), glucose, and LA concentrations. To determine the best nitrogen sources for LA production, different concentrations of yeast extract (0, 2.5, 5.0, 7.5, 10.0 g L^−1^), peptone (0, 5.0, 10.0, 15.0, 20.0, 25.0 g L^−1^), and beef extract (0, 5.0, 10.0, 15.0, 20.0, 25.0 g L^−1^) were supplemented separately in 250 mL-Erlenmeyer flasks containing 100 mL of the media described above at 37°C, pH controlled at 9.0 for 30 h. Samples were withdrawn periodically for the analysis of growth and fermentation products. To determine the optimal temperature, media were prepared and inoculated at 10% of preculture broth. Fermentation processes were conducted at 25, 30, 37, 40, 45, 50, 55, and 60°C.

### 2.4. Fed Batch and Repeated Batch Fermentation

Multipulse fed batch fermentation was conducted in a 1.0-l jar fermentor with a working volume of 300 mL of optimized MRS medium using* E. hirae* BoM1-2. Fermentation was initiated by cell inoculation at 10% (*v/v*). pH was controlled at 9.0 by 10 N of NaOH at 40°C. Samples were taken every 3-6 h for the analysis of cell growth (OD_600nm_), glucose, and LA concentrations. Intermitted feeding using glucose (30 or 40 g L^−1^) and yeast extract (1 g L^−1^) was added to the fermentor when the residual glucose concentration reached 10 g L^−1^. Feeding was supplemented after 36 h (40 g L^−1^), 69 h (40 g L^−1^), 138 h (30 g L^−1^), and 204 h (30 g L^−1^) of fermentation. Repeated batch fermentation processes were performed using 300 mL in 1L jar fermentor supplemented with the desired glucose concentrations at 40°C. pH was controlled at 9.0 using NaOH. At the end of fermentation processes, fermentation broth was centrifuged (6000 rpm for 10 min at 5°C), and the cells were resuspended in new fresh media to perform next batch (run). Two fermentation strategies were conducted. In the first strategy, fermentation processes were conducted for eleven runs using optimized MRS medium supplemented with 60 g L^−1^ glucose in the first 10 runs and 100 g L^−1^ glucose in the last run. In the second strategy, fermentation processes were conducted for 24 runs. At the first 22 runs, optimized MRS media were used with different initial glucose concentrations. Runs 1–13 were supplemented with 40 g L^−1^ glucose; runs 14–16 were supplemented with 60 g L^−1^ glucose; runs 17–19 were supplemented with 80 g L^−1^ glucose; runs 20-22 were supplemented with 100 g L^−1^ glucose. Runs 23-24 were supplemented with 100 g L^−1^ glucose but exclusion of peptone from medium composition was carried out in 23rd run, while peptone and beef extract were excluded in 24th run.

### 2.5. Analytical Methods and Fermentation Parameters

Cell growth was estimated based on OD_600nm_ measurements using a visible spectrophotometer. Fermentation samples were centrifuged at 6,000 rpm (4,427*xg*) for 10 min at 4°C and the supernatants were analyzed for glucose and LA determination. Residual glucose in the broth was estimated by using 3, 5-dinitrosalicylic acid reagent (DNS method) [14]. LA was measured by the modified Barker and Summerson method [15] and confirmed with HPLC analysis as described previously [10]. The concentrations of residual glucose and LA were calculated using calibration curves obtained using the standard solutions. The yield of LA (Y, g g^−1^) was defined as the ratio of lactic acid produced (g L^−1^) to amount of glucose consumed (g L^−1^). LA productivity (g L^−1 ^h^−1^) was calculated as the ratio of lactic acid concentration (g L^−1^) to the fermentation time (h) at which the maximum lactic acid concentration was obtained. Maximum productivity was calculated between each sampling period within exponential growth phase. All experiments were conducted in triplicate, and data were statistically analyzed by SPSS v17 (SPSS Inc., Chicago, IL, USA). One-way analysis of variance (ANOVA) test was used for sample comparison when normality and homogeneity of variance were satisfied and followed by multiple comparison using Tukey's range tests at* P*<0.05.

## 3. Results

### 3.1. Isolation and Characterization of Alkaliphilic Lactic Acid Bacteria

A total of 369 purified bacterial isolates were obtained from the 27 different sources collected from Egypt. Among them, 80 bacterial isolates were primarily considered as lactic acid bacteria that showed catalase negative activity and yellow color around the colonies on MRS plates containing bromocresol green. These isolates were cultured in broth media for quantitative determination of LA. Out of 34 isolates that produced LA > 2 g L^−1^, only 17 isolates were selected because these isolates produced LA higher than 5 g L^−1^ with high yield > 0.80 g g^−1^-consumed sugars as shown in Fig.  S1 (see supplementary data). These isolates also exhibited better tolerance to high glucose concentration (up to 100 g L^−1^) and to acetic acid (up to 20 g L^−1^) [data not shown]. Among them, the isolate BoM1-2, which was obtained from milk sample collected from Beheira governorate at Egypt, was described as the highest lactic acid producer. This isolate produced 9.1 g L^−1^ of LA with high LA yield (0.95 g g^−1^ of glucose consumed) and showed the highest growth at OD_600nm_ of 0.887.

**Figure 1:**
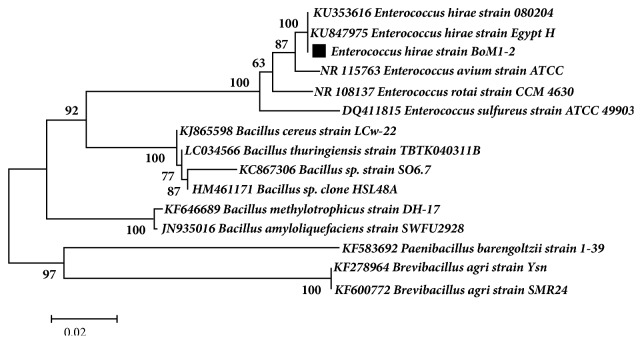
Phylogenetic tree based on 16S rRNA gene sequences. The evolutionary history was inferred by using the maximum likelihood method based on the Kimura two-parameter model. The percentage of replicate trees in which the associated taxa clustered together in the bootstrap test (1000 replicates) is shown next to the branches. Black squares indicate the organisms isolated in this study. GenBank accession numbers of reference sequences are indicated. Bar 0.02 nucleotide substitutions per position.

The selected isolate BoM1-2 was characterized by biochemical and molecular methods. As indicated in Table S1 (see supplementary data), isolate BoM1-2 is Gram positive, of coccus shape, catalase negative, and facultative anaerobic. BoM1-2 produced l-lactic acid homofermentatively at an optical purity of 99.7%. The colonies are white in color and convex on agar plates. This isolate showed high growth at a wide range of temperature 25–60°C and had the ability to grow at broad spectrum of pH values, 5.0–10.0. On the other hand, this isolate could not utilize citrate, urea, pectin, starch, cellulose, or gelatine. Moreover, isolate BoM1-2 showed no blood haemolysis on agar plates, which showed the safety of this isolate. BoM1-2 can grow under high concentration of sodium chloride up to 7.5%. The isolate was also characterized by bacterial identification kit of API- 50 CHL, which tested the acid formation from various sugars and sugar alcohol. As shown in Table S2, the sugar fermentation pattern of strain BoM1-2 excluding utilization of l-arabinose, d-mannitol, and amygdaline showed the highest similarity to those of* Enterococcus hirae* among those of enterococcal species described by Manero and Blanch [16]. The molecular identification of 16S rRNA gene fragments of this isolate showed that it was identified as* Enterococcus hirae* with 99% identity to* Enterococcus hirae* strain Egypt H (GenBank accession number of KU847975) and* Enterococcus hirae* strain 080204 (GenBank accession number of KU353616). Phylogenetic analysis based on 16S rRNA gene sequencing and alignment revealed that isolate BoM1-2 was clustered with* Enterococcus hirae *strains with supported bootstrap value of 96 (Figure 1).* Enterococcus hirae* strain BoM 1-2 16S ribosomal RNA gene has been deposited in NCBI GenBank under the accession number MK026806 (https://www.ncbi.nlm.nih.gov/search/all/?term=mk026806).

### 3.2. Optimization of Fermentation Conditions

#### 3.2.1. Effect of Various pH Values and Nitrogen Sources on Lactic Acid Production

To investigate the influence of pH on lactic acid production by* E. hirae* BoM1-2, fermentation processes were conducted at various initial pH values (5.0–11.0) using the modified MRS media supplemented with 20 g L^−1^ glucose (Table 1). Strain BoM1-2 showed comparable growth at pH 5.0–10.0 with OD_600nm_ range of 0.930–1.04, while decreased growth of 0.620 was obtained at pH 11.0 with drastic decrease in glucose consumption (3.6 g L^−1^) and LA production (3.0 g L^−1^). The strain exhibited homofermentative LA production at all tested pH values with LA yields of 0.83–0.90 g g^−1^-consumed glucose. The highest glucose consumption of (15.4–15.6 g L^−1^), LA production (13.9 g L^−1^), and maximum LA productivities (1.9 g L^−1 ^h^−1^) were obtained at pH 8.5-9.0. Higher yield at 0.90 g g^−1^-consumed sugar was obtained at pH 9.0 and therefore was selected as the optimal fermentation condition for further experiments.

Different concentrations of nitrogen sources including yeast extract (0–10 g L^−1^), peptone (0–25 g L^−1^), and beef extract (0–25 g L^−1^) were supplemented to modified MRS media to determine the best medium composition for BoM1-2 using 20 g L^−1^ glucose at pH 9.0 and 37°C. The highest OD_600nm_ (1.13), sugar consumption (18.8 g L^−1^), LA concentration (17.0 g L^−1^), LA productivity (0.57 g L^−1 ^h^−1^), and maximum LA productivity (2.9 g L^−1 ^h^−1^) were obtained in medium contained 7.5 g L^−1^ yeast extract, 20 g L^−1^ peptone, and 20 g L^−1^ of beef extract (data not shown), that is, 22% increment in LA concentration compared to control. In addition, supplementation of inorganic nitrogen sources did not exhibited effect on increment of LA production.

#### 3.2.2. Effect of Different Temperatures on Lactic Acid Production

The effect of temperature on the growth and production of lactic acid by* Enterococcus hirae* was studied in the range of 25-60°C and pH 9.0 for 30 h (Table 2). Strain BoM1-2 exhibited comparable growth pattern at all tested temperatures that record high OD_600nm_ ranging from 0.97 to 1.05 with the highest value at 40–45°C. Comparable glucose consumption was also achieved ranging between 18.7 and 19.7 g L^−1^ at wide range of temperature (25–55°C) with the maximum value of 19.7 g L^−1^ at 40°C. On the other hand, decreased glucose consumption of 15.2 g L^−1^ was obtained at 60°C. Similar pattern was exhibited for LA production within the range 17.1–18.4 g L^−1^ at 25–55°C with the highest value of 18.4 g L^−1^ at 40°C. Decreased LA production of 13.2 g L^−1^ was obtained at 60°C. Maximum LA yield of 0.93 g g^−1^-glucose consumed was obtained at 40°C with a maximum LA productivity of 3.1 g L^−1 ^h^−1^ and total productivity of 0.61 g L^−1 ^h^−1^, so that 40°C was selected as the optimal temperature for further studies.

#### 3.2.3. Effect of Different Glucose Concentrations on Lactic Acid Production

**Figure 2:**
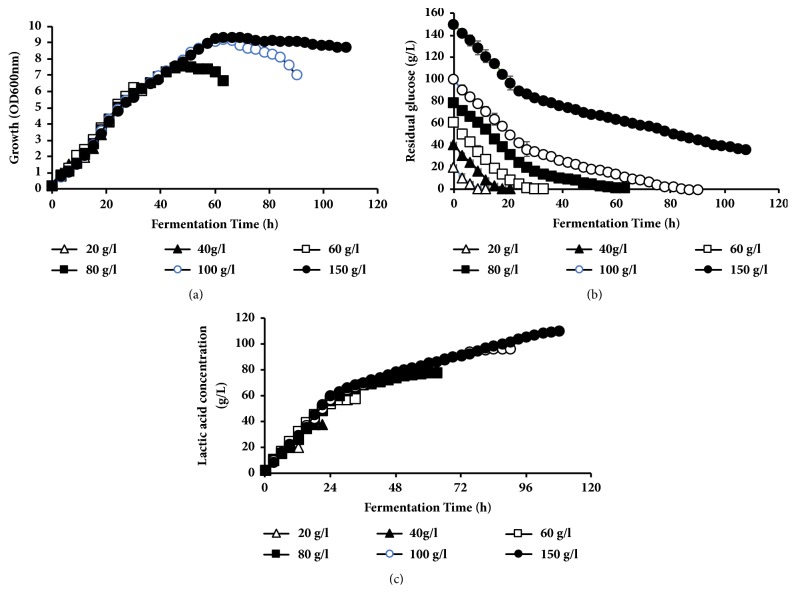
Profiles of lactic acid fermentation using different glucose concentrations by* E. hirae* BoM1-2. (a) Growth [OD_600nm_]. (b) Residual glucose concentration [g L^−1^]. (c) Lactic acid concentration [g L^−1^].

To investigate the effect of glucose concentration on lactic acid production, fermentation processes were conducted with different initial glucose concentrations (20–150 g L^−1^) at pH controlled at 9.0 (Table 3; Figure 2). Strain BoM1-2 exhibited short lag phase at all tested concentrations with final biomass increment from OD_600nm_ of 1.99 at initial 20 g L^−1^ glucose up to OD_600nm_ of 9.72 at initial 150 g L^−1^ glucose (Figure 2(a)). The strain exhibited long stationary phase at 80–150 g L^−1^ glucose while glucose consumption and LA production was achieved. Glucose was completely consumed at an initial glucose concentration 20–100 g L^−1^ with production of 19.6–96.0 g L^−1^ lactic acid at high yield ranging from 0.93 to 0.96 g g^−1^-consumed glucose (Figures 2(b) and 2(c)). On the other hand, 35.9 g L^−1^ of glucose remained in fermentation medium after 108 h in fermentation processes with 150 g L^−1^ glucose with maximum LA production of 109.9 g L^−1^. LA productivities were decreased with an increase of glucose concentration achieving the highest value of 2.18 g L^−1 ^h^−1^ in fermentation processes using 20 g L^−1^ glucose and 1.02 g L^−1 ^h^−1^ in fermentation processes using 150 g L^−1^ glucose, while the maximal LA productivities were almost comparable at fermentation processes with 20–100 g L^−1^ glucose within the range 3.1–3.4 g L^−1 ^h^−1^. The highest value for maximal LA productivity of 3.4 g L^−1 ^h^−1^ was obtained from 60 g L^−1^ glucose (Table 3).

### 3.3. Fed Batch Fermentation for Lactic Acid Production

**Figure 3:**
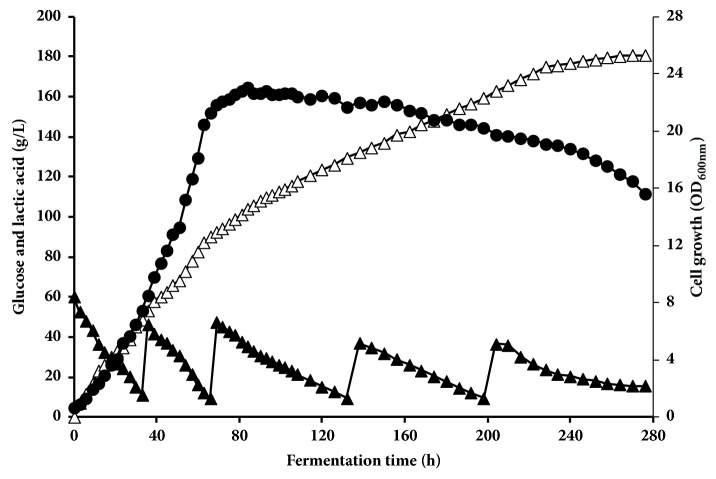
Fed batch fermentation for lactic acid production by BoM1-2 using medium containing initial 60 g L^−1^ glucose at 40°C. Symbols: ▲, residual glucose (g L^−1^); △, LA (g L^−1^); ●, optical density at 600 nm.

In a trial to increase LA production and productivity, multipulse fed batch strategy was conducted with an initial glucose concentration of 60 g L^−1^ and feed with 40 g L^−1^ of glucose and 1 g L^−1^ of YE when the glucose concentration reached to about 10 g L^−1^ until the total added glucose reached 140 g L^−1^; after that 30 g L^−1^ of glucose and 1 g L^−1^ of YE were added until the glucose concentration reached 200 g L^−1^. As shown in Figure 3, the growth of the BoM1-2 strain was increased with time achieving maximum OD_600nm_ of 22.03 at 72 h, became almost stable for another 90 h, and then decreased gradually to OD_600nm_ of 15.6 at the end of fermentation (276 h). Glucose consumption rate was gradually decreased after 72 h of fermentation that is in accordance with LA production rate. A final LA concentration of 180.6 g L^−1^ was obtained with a yield of 0.98 g g^−1^-consumed glucose at LA productivity of 0.65 g L^−1 ^h^−1^ and residual glucose concentration of 15.6 g L^−1^. Therefore, fed batch fermentation increased fermentation parameters in terms of sugar consumption and LA production compared to batch mode while resulting in decreased LA productivity.

### 3.4. Different Repeated Batch Fermentation Strategies

**Figure 4:**
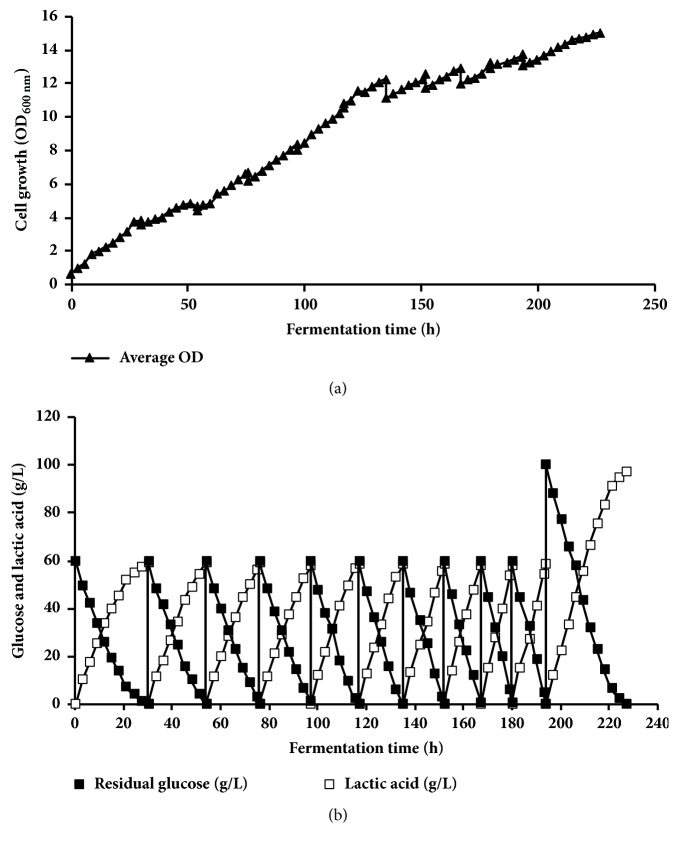
Lactic acid production by* E. hirae *BoM 1-2 in repeated batch fermentation (1–11 runs) with initial glucose concentration of 60 g L^−1^ at the first 10 runs and 100 g L^−1^ at run 11, pH 9.0 at 40°C. (a) Cell growth. (b) Residual glucose and lactic acid concentration. Symbols: ▲, OD; ■, residual sugar; □, lactic acid.

Repeated batch fermentation processes using all recycled cells were conducted with an initial glucose concentration of 60 g L^−1^ for the first 10 runs and 100 g L^−1^ glucose for the last run (11th run) (Figures 4(a) and 4(b)). The growth of BoM1-2 strain was increased with subsequent runs achieving a maximum OD_600nm_ of 3.8 in first run and OD_600nm_ of 13.7 at the 10th run. Fermentation time was greatly decreased from 30 h at the 1st run to 13 h at 9th and 10th runs with comparable LA concentrations (58.8-60.0 g L^−1^) and LA yields (0.97-0.99 g g^−1^-consumed glucose). LA productivity was increased from 1.95 g L^−1 ^h^−1^ at the 1st run up to 4.48 g L^−1 ^h^−1^ at 10th run (Table 4). Increasing glucose concentration to 100 g L^−1^ at 11th run has resulted in long fermentation time (33h) with production of 97.2 g L^−1^ of LA at yield of 0.97 g g^−1^-consumed glucose and LA productivity of 2.95 g L^−1 ^h^−1^ (Table 4).

**Figure 5:**
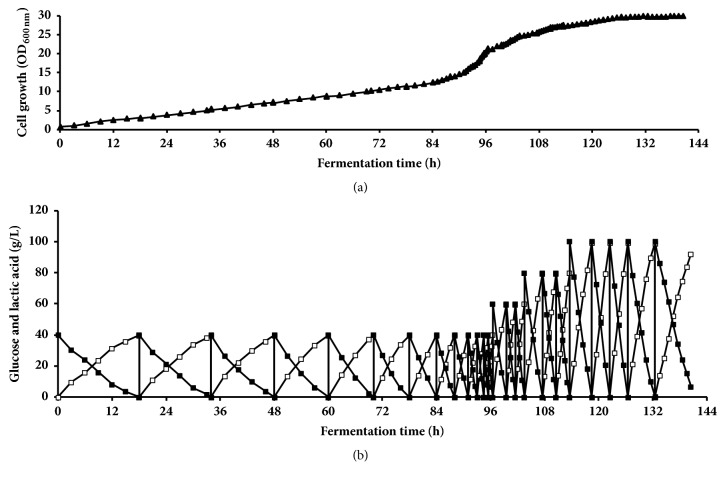
Repeated batch fermentation (1–24 runs) for lactic acid production by BoM1-2 using medium containing initial 40–100 g L^−1^ glucose at 40°C, pH controlled at 9.0. (a) Cell growth profile. (b) Glucose consumption and lactic acid production. Symbols: ▲, average optical density; OD at 600 nm; ■, glucose concentration; □, produced lactic acid.

In a rational trial for improved LA productivity, repeated batch fermentation processes were conducted with initial glucose concentration of 40 g L^−1^ for the first 13 runs, and then glucose concentration was gradually increased in subsequent runs [60 g L^−1^ at runs 14th-16th; 80 g L^−1^ at runs 17th–19th; and 100 g L^−1^ at runs 20th–24th] (Table 5, Figure 5). The data recorded in Table 5 summarized the kinetic parameters of all runs. Strain BoM1-2 showed an increased growth with repeated cycles from OD_600nm_ 3.1 at the 1st run up to 21.2 at 13th runs. At these runs, glucose was completely consumed with comparable LA production that ranged from 39.6 to 39.9 g L^−1^ at LA yield of 0.99–1.0 g g^−1^-consumed sugar. In the 1st run, fermentation lasted after 18 h; after that, the fermentation time was significantly decreased at the subsequent runs to achieve 18-fold decrease (only 1 h) at 12th and 13th run. Significant increase in LA productivity (18-fold) was obtained, which achieved 39.9 g L^−1 ^h^−1^ at the 13th run compared to 2.2 g L^−1 ^h^−1^ at the 1st run.

To increase the final lactic acid concentration, 14th-16th runs were conducted in the same MRS media supplemented with 60 g L^−1^ glucose. At the 14th run, the growth was further increased to achieve OD_600nm_ of 22.3, and complete consumption of glucose was achieved with production of LA at 59.7 g L^−1^ at LA yield of 1.0 g g^−1^-glucose consumed. On the other hand, fermentation time was increased to 3 h, which achieved lower LA productivity of 19.9 g L^−1 ^h^−1^ compared to previous runs. Similar lactic acid concentration (59.4–59.8 g L^−1^) and LA yield (0.99-1.0 g g^−1^ of glucose consumed) were obtained in the 15th and 16th run, while shorter fermentation time (2 h) and higher LA productivity (29.7 g L^−1 ^h^−1^) were obtained. Almost comparable results were obtained in the 16th run as 15th run with little increase in OD_600nm_ (24.7) (Table 5).

In a further attempt to increase the lactic acid concentration with the same components of MRS media, 17th–19th runs were conducted with higher concentration of 80 g L^−1^ glucose. As shown in Figure 5, OD_600nm_ was increased within the range 25.5–27.4 in these runs with complete consumption of glucose. LA concentration was increased to achieve 79.4–79.6 g L^−1^ with high LA yield that ranged from 0.99 to 1.0 g g^−1^. On the other hand, decreased LA productivity was obtained at 17th run (19.8 g L^−1 ^h^−1^) compared to the previous 16th run (29.9 g L^−1 ^h^−1^), while it became increased in subsequent runs to achieve 26.47–26.53 g L^−1 ^h^−1^ in run 18th and 19th with short fermentation time of only 3 h.

To further increase the LA concentration, the 20th–22nd runs were conducted using 100 g L^−1^ of glucose with the same media components. Growth and LA concentration were increased to record maximal value of 28.1 and 99.1 g L^−1^, respectively, after 5 h of fermentation time, while LA productivity decreased to 19.8 g L^−1 ^h^−1^ compared to the previous, 19th, run (26.5 g L^−1 ^h^−1^). On the subsequent run (21st), the increasing growth (OD_600nm_ of 28.9) resulted in increasing LA productivity to 24.8 g L^−1 ^h^−1^ with almost the same LA concentration. Similar results were obtained at 22nd run with little increase of growth recording maximal OD_600nm_ of 29.6 with fermentation time of only 4 h.

To decrease the production cost for LA fermentation by BoM1-2 strain, reducing of nitrogen source in media was achieved through excluding the peptone from MRS media in 23rd run. Growth, LA concentration, and LA yield were almost similar to previous run at 29.7, 98.8 g L^−1^, and 0.99 g g^−1^ of glucose consumed, respectively, compared to 22nd (containing peptone) at 29.6, 99.2 g L^−1^, and 0.99 g g^−1^ of glucose consumed, respectively. On the other hand, fermentation proceeded for longer time (6 h) than 4 h in 22nd run, which resulted in decrease of LA productivity to 16.47 g L^−1 ^h^−1^ compared to 24.8 g L^−1 ^h^−1^ achieved in the previous run. Exclusion of peptone and beef extract in 24th run [medium contained only 7.5 g L^−1^ of YE as the sole nitrogen source] resulted in a decrease in LA acid concentration with only 92.0 g L^−1^ (compared to 98.8 g L^−1^ of previous run), and LA productivity of 11.5 g L^−1 ^h^−1^ compared to 16.5 g L^−1 ^h^−1^ was obtained. On the other hand, almost no changes in the cell growth and LA yield at 29.8 and 0.99 g g^−1^ of glucose consumed, respectively, were obtained as compared to previous runs. From the above results, repeated batch fermentation by* E. hirae* BoM1-2 coupled with increased glucose concentration strain achieved gradual increase in cell growth and greatly improved LA productivity as compared to fermentation in batch modes using respective sugar concentrations.

## 4. Discussion

Lactic acid (LA) and its derivatives are among the most important products having applications in many industries [9]. LA can be produced by various microorganisms including bacteria, fungi, and microalgae; however, bacterial strains are preferred over other microorganisms because of their fast growth and high productivities [8]. For commercial applications, robust microbes that can meet the industrial needs for high LA titre, productivity, yield, and high optical purity are required. In addition, overcoming some production challenges such as contamination risk by neutrophilic microorganisms and reduction of neutralizing agents is of great importance [1]. Consequently, new microbial isolates with enhanced LA production capabilities are needed to reduce the production and purification costs. Therefore, the present study aimed to isolate and characterize potential alkaliphilic LAB and improve the production and productivity by using different modes of fermentation.

In this study, 369 bacterial isolates were obtained from different environmental samples collected from different localities in Egypt; among those, 80 isolates produced acid on agar medium and showed no catalase activity that preliminary considered as LAB. Quantitative determination of LA, investigation of high glucose tolerance, and high acetic acid tolerance indicated that isolate BoM 1-2 is considered as the most potent LAB producer based on LA production, LA yield, and homofermentative LA production pattern. Morphological and biochemical characterization revealed that this isolate was similar to* Enterococcus* spp. (Tables S1 and S2, see supplementary data). Molecular identification depending on 16S rRNA gene fragments amplifications and sequencing confirmed that this isolate was identified as* Enterococcus hirae* BoM1-2. Natural sources are always the most powerful means for obtaining useful and genetically stable strains for industrially important products [17]. Several enterococci strains have been isolated from natural resources such as thermophilic* E. faecium* QU 50 [17] and* E. faecalis*CBRD01 [18] that were isolated from soil sample, and* E. mundtii* QU 25 that was isolated from bovine fecal samples [19].

To improve LA production in* E. hirae* BoM 1-2, fermentation parameters including pH, medium composition, temperature, and initial sugar concentrations were varied and modified.* E. hirae* BoM1-2 strain showed better growth, sugar consumption, LA production, LA productivity, and LA yield at alkaline pH values of 8.5–9.0. On the other hand, low pH (less than 7.0) and highly alkaline pH (more than 10.0) had negative effect on the growth of strain BoM1-2 which eventually reflected in terms of less metabolites production. This might be due to the enzyme denaturation at these conditions. These results indicated that strain BoM 1-2 is alkaliphilic, that is, more advantageous for LA production than commonly used LAB. Alkaliphilic strains can tolerate high levels of salts and consequently reduce the risk of contamination and decrease the amount of required neutralizing agents [4]. This is the first study to report on alkaliphilic production of LA using* E. hirae*. Previously reported* Enterococcus* strains achieved LA fermentation at neutral or slightly acidic conditions. Abdel-Rahman et al. [10] reported that the optimal pH for LA production from glucose in batch fermentation by* E. mundtii* QU 25 was 7.0. Value of 6.5 was considered as the optimal pH for LA production by* E. faecium* using xylose [17] or starch [20] in batch fermentation.

LAB bacteria are fastidious organisms, which need complex nutrients as amino acids and vitamins for cell growth [21]; therefore, it is necessary to determine the optimal media composition for the selected strains. Different concentrations of nitrogen sources were tested at pH 9.0. The optimal concentrations of yeast extract, peptone, and beef extract were 7.5 g L^−1^, 20 g L^−1^, and 20 g L^−1^, respectively, which achieved the highest fermentation efficiency. Yeast extract, peptone, and beef extract supplement contain critical amounts of vitamins, and trace elements are essential for growth and LA biosynthesis [22]. Higher concentrations led to decrease of all fermentation parameters indicating that these high concentrations might be toxic [22]. Also, the absence of any of these nitrogen sources decreases the fermentation efficiency and leads to weak growth due to nutritional limitations and deficiency in peptide sources (amino acids) or in growth factors. Dumbrepatil et al. [23] reported that YE at 10 g L^−1^ is the optimum concentration for LA production from corncob molasses. Chiarini et al. [24] reported that a maximum LA concentration by* Lb. helveticus *using whey permeate provided with 15–25 g L^−1^ yeast extract. Also, Wee et al. [25] found that the optimal concentration of YE that achieved the highest productivity and dry cell weight for* E. faecalis* RKY1 was 20 g L^−1^, while Kotzamanidis et al. [22] reported that the 50 g L^−1^ YE was the optimum concentration of YE for all fermentation parameters by* L. delbrueckii* NCIMB 8130 using beet molasses as the sole carbon source.

It is important to maintain the operating temperature at the optimal level because it affects the rate of growth, enzyme activities, biochemical reactions as well as substrate consumption rate, and LA production efficiency [2, 17, 19]. In this study,* E. hirae* BoM1-2 produced LA at a wide range of temperature 25–55°C, with the optimal fermentation at 40°C. Higher temperature over 55°C adversely affected the LA fermentation. This might be due to the decreased biochemical reactions rates, generation time, substrate conversion rate, and the enzymatic activities catalysing LA production [17]. Most of the lactic acid-producing enterococci described in the literature show maximum production of lactic acid in the temperature range of 30-43°C. Sun et al. [26] found that 40°C was the optimal temperature for LA production by* E. faecium. *Nandasana and Kumar [27] reported that 38°C was the optimal temperature for LA production by* E. faecalis *RKY1, while Abdel-Rahman et al. [10, 19, 28] reported that maximum LA production from cellobiose, glucose, and xylose occurred at 43°C by* E. mundtii* QU 25. Shibata et al. [20] found that 30°C was the optimal temperature for the production of lactic acid from sago starch by* E. faecalis*. In contrast, Abdel-Rahman et al. [17] found that 50°C was the optimal temperature for the production of lactic acid from xylose by* E. faecium* QU 50.

For commercial production of LA, high LA concentration, yield, and productivity are important key fermentation parameters to reduce the production cost. The high initial substrate tolerance is very important for getting high LA production to reduce the downstream processing costs [1, 8]. Interestingly, fermentation by BoM1-2 strain under pH control at 9.0 showed increased biomass with an increase of initial glucose concentration from 1.99 OD_600nm_ at initial 20 g L^−1^ of glucose up to 9.72 OD_600nm_ at 150 g L^−1^ glucose with homofermentative production of LA at yield range of 0.90-0.98 g g^−1^ of glucose consumed. This indicates that strain BoM1-2 exhibited osmotolerance at high glucose concentrations, which offers potential advantages in fermentative LA production enabling high production and facilitating downstream processing. This also indicates that substrate inhibition may be negligible because it showed a short initial lag phase followed by a rapid growth phase and the growth is not suppressed at all tested concentrations. Stationary phase appears to be long at high initial concentration of glucose (80-150 g L^−1^) after the cell mass reached the maximum value as a result of accumulation of LA and/or nutrient exhaustion. On the other hand, LA was still produced as glucose metabolized but led to decreased LA productivity at high glucose concentration due to the increase of fermentation time. The maximum LA productivity of 2.18 g L^−1 ^h^−1^ was achieved at 20 g L^−1^ glucose and decreased by increasing glucose concentration. Moreover, the residual sugar was also increased as a result of LA accumulation in the media that might indicate end product inhibition. LA fermentation efficiency by strain BoM1-2 using glucose was mostly comparable to the LA concentration, LA yield, and LA productivity obtained using cellobiose (89.8 g /L, 0.913 g g^−1^, and 1.25 g L^−1 ^h^−1^, respectively) and xylose (86.6 g L^−1^, 0.83 g g^−1^, and 0.902 g L^−1 ^h^−1^, respectively) [19, 28] from 100 g L^−1^ of each sugar in batch fermentation. Our results are in agreement with other reports showing increased LA concentration with the increase of initial glucose concentrations, while very high concentrations of sugar resulted in increasing osmotic pressure and decreasing LA productivity [10, 11].

The challenge to increase LA concentration and LA productivity from high glucose concentrations needs to be addressed for commercial LA production. We conducted multipulse fed batch fermentation to reduce the osmotic stress of high initial sugar on microbial growth achieved in the batch culture condition that might combine with plasmolysis and result in a decrease in the rate of fermentation and sugar utilization. A high increase in cell biomass achieved a maximum value of 22.03 [OD_600nm_] at 72 h of fermentation. A final LA concentration of 180.6 g L^−1^ was obtained after 276 h of fermentation. This led to decreasing the total LA productivity recording 0.65 g L^−1 ^h^−1^ that is lower than that obtained by batch fermentation. No by-products were detected at all fermentation time with LA yield at 0.98 g g^−1^-glucose consumed. Results obtained in the current study under alkalophilic condition were superior to other LAB strains reported so far. Yoshimune et al. [29] achieved a maximum LA production of 149 g L^−1^ and 153 g L^−1^ from 180 g L^−1^ glucose at pH 9.0 and 35°C by* E. casseliflavus* L-120 and* E. faecalis *AY103, respectively. A maximum LA concentration of 103 g L−1 and LA yield of 0.8 g g^−1^ were from 129 g L^−1^ of glucose by* E. casseliflavus* strain 79w3 at pH 8.0 in batch fermentation [6].* Exiguobacterium *sp. 8-11-1 produced 125 g L^−1^ of LA at pH 8.5 and 37°C in fed batch fermentation [5].

The repeated batch process involves repeated cycles of fermentation by reinoculating a part or all of the cells from one batch fermentation broth into the next batch fermentation medium [30]. In comparison with batch or fed batch culture, repeated batch operation has proved to have several advantages in increasing LA productivity besides saving the time and labor work [30]. BoM1-2 strain was firstly cultivated in repeated batch fermentation using 60 g L^−1^ of glucose for 10 runs at controlled pH 9.0. Increased cell mass was obtained from OD of 3.8 at the 1st run up to 13.7 at 10th run that consequently resulted in increase of LA productivity from 1.97 g L^−1 ^h^−1^ at 1st run to 4.48 g L^−1 ^h^−1^ at 10th run (2.27-fold), while the final LA concentration and LA yield remain at similar value. This improvement of LA productivity was attributed to increased cell biomass. In the 11th run, the fermentation with 100 g L^−1^ of glucose resulted in little increase in biomass to OD of 15.0 with LA concentration of 97.2 g L^−1^ at yield of 0.97 g g^−1^ of glucose consumed while decrease in LA productivity of 2.95 g L^−1 ^h^−1^ was obtained as compared to 4.48 g L^−1 ^h^−1^ obtained in the previous run.

In another strategy, we conducted repeated batch fermentation processes coupled with gradual increase of glucose concentrations in the range 40-100 g L^−1^ as a rational trial for cell adaption to higher sugar concentrations via 24-run fermentation. High increase in cell biomass (OD_600nm_ of 21.2) was obtained at 13th run using 40 g L^−1^ glucose. This resulted in a significant increase in the LA productivity compared to previous experiment, where LA productivity increased by 18-fold from 2.2 g L^−1 ^h^−1^ at 1st run to 39.9 g L^−1 ^h^−1^ at 13th run. In the subsequent three runs using 60 g L^−1^ glucose, cell biomass was further increased, resulting in increased LA concentration (59.7 g L^−1^) with LA productivity of 29.9 g L^−1 ^h^−1^ compared to LA productivity of 1.75 obtained in normal batch fermentation (Table 3). For further increase of LA concentration, the three subsequent runs were conducted using initial 80 g L^−1^ of glucose, and then another three runs were performed with 100 g L^−1^ of glucose. Interestingly, the strain showed the same behavior as that obtained with previous runs of increased cell biomass, complete glucose consumption, and increased LA production as expected. LA productivity was 26.4 and 24.8 g L^−1 ^h^−1^ using 80 and 100 g L^−1^ of glucose, respectively, compared to 1.23 and 1.07 g L^−1 ^h^−1^ obtained in normal batch fermentation (Table 3), that is, about 23- and 21-fold, respectively. This significant increment of LA productivity of about 17-23 was not reported previously in literature for LA production. Although decreased cell growth was obtained with nitrogen source exclusion in subsequent runs (23th and 24th), LA productivity was 11.5-16.5 g L^−1 ^h^−1^, which is also higher than results obtained in normal batch fermentation with complete medium (1.07 g L^−1 ^h^−1^; Table 3). This superior improvement might be due to the adaption of cultivated cells to higher sugar concentrations and increasing the potency and efficiency of microbial cells for substrate conversion and LA production. This indicated that our new strategy is a significant finding for improved green chemical productivity. To the best of our knowledge, this study is not only the first study to report on the LA production by repeated batch fermentation under alkaliphilic conditions, but also the first study to report very high LA productivity (39.9 g L^−1 ^h^−1^) and to achieve 24 runs for repeated batch in LA fermentation. Therefore, we succeeded in establishing a highly productive system for obtaining LA at high concentration and high productivity in repeated batch fermentation for at least 24 runs within 140.5 h that would greatly reduce the production cost of LA. There are only a few reports that related LA production for using repeated batch fermentation by LAB. Abdel-Rahman et al. [10] achieved the highest LA productivity of 16.7 g L^−1 ^h^−1^ by* E. mundtii* QU 25. Oh et al. [21] achieved the highest LA productivity of 6.03–6.20 g L^−1^ by* E. faecalis* RKY1 with 100 g L^−1^ glucose. The highest LA productivity of 4.0 g L^−1 ^h^−1^ was obtained by* E. faecalis* RKY1 from wood hydrolyzate containing 50 g L^−1^ glucose [31]. Kim et al. [32] obtained LA productivity at 6.34 g L^−1 ^h^−1^ from 100 g L^−1^ lactose in cheese whey by* Lactobacillus* sp. RKY2 through the cell-recycle repeated batch fermentation.

In conclusion, efficient production of lactic acid from alkaliphilic wild type* E. hirae *BoM1-2 was obtained. Its alkaliphilic characteristics could reduce the risk of contamination during fermentation process and using NaOH as neutralizing agent allowed for cleaner processing. Batch fermentation exhibited limited lactic acid productivity of 2.18 g L^−1 ^h^−1^ as a maximum total LA productivity at pH 9.0 and 40°C. Lactic acid concentration was substantially enhanced by fed batch fermentation that reached 180.6 g L^−1^. Repeated batch fermentation processes were performed for 24 runs without any loss in the fermentation efficiency. Lactic acid productivity and maximal productivity reached the highest values in lactic acid fermentation technique, 39.9 g L^−1 ^h^−1^ and 49.7 g L^−1 ^h^−1^, respectively, which resulted in a considerable decrease in the fermentation time as a result of significant increase of culture biomass. Therefore, we could establish a highly efficient system for the production of polymer-grade lactic acid using* E. hirae* BoM1-2, a strong candidate for industrial production of lactic acid, with superior LA productivity* via* coupling increased sugar concentration during repeated fermentation.

## Figures and Tables

**Table 1:** Effect of different pH values on the growth and kinetic parameters of lactic acid production from glucose by *Enterococcus hirae *BoM1-2.

**pH value**	**O** **D**_600 **nm**_^**a**^	**Consumed glucose (g L** ^**−1**^ **)**	**LA conc. (g L** ^**−1**^ **)** ^**b**^	**Y**_**LA**_ ** (g g** ^**−1**^ **)** ^**c**^	**P**_**LA**_ ** (g L** ^**−1**^ ** h** ^**−1**^ **)** ^**d**^	**Max. ** **P**_**LA**_ ** (g L** ^**−1**^ ** h** ^**−1**^ **)** ^**e**^
**5.0**	0.930±0.05	6.80±0.1	6.10±0.1^e^	0.90	0.20^e^	0.80(6 h)
**6.0**	0.970±0.03	8.50±0.3	7.60±0.2^d^	0.89	0.25^d^	0.90(6 h)
**7.0**	1.01±0.01	9.80±0.1	8.80±0.1^c^	0.90	0.29^c^	1.60 (3 h)
**8.0**	1.01±0.01	12.1±0.2	10.9±0.2^b^	0.90	0.36^b^	1.80 (3 h)
**8.5**	1.03±0.01	15.6±0.2	13.9±0.1^a^	0.89	0.46^a^	1.90 (3 h)
**9.0**	1.04±.0.06	15.4±0.3	13.9±0.1^a^	0.90	0.46^a^	1.9 (3 h)
**10.0**	0.980±0.05	10.1±0.1	9.0±0.0^c^	0.89	0.30^c^	0.80(3 h)
**11.0**	0.620±0.02	3.60±0.1	3.0±0.0^f^	0.83	0.10^f^	0.20(6 h)

^a^OD, maximum optical density. ^b^ Maximum lactic acid concentration after 30 h. ^c^ Lactic acid yield. ^d^ Lactic acid productivity at the end of fermentation time. ^e^ Maximum lactic acid productivity at indicated time. Different letters between columns denote that mean values are significantly different (p≤0.05) by Tukey test; values are means ± SE (n=3).

**Table 2:** Effect of different temperatures on the growth and kinetic parameters of lactic acid production from glucose by *Enterococcus hirae *BoM1-2.

**Temperature ** **(**°**C)**	**O** **D**_600 **nm**_^**a**^	**Consumed glucose (g L** ^**−1**^ **)**	**LA conc. (g L** ^**−1**^ **)** ^**b**^	**Y**_**LA**_ ** (g g** ^**−1**^ **)** ^**c**^	**P**_**LA**_ ** (g L** ^**−1**^ ** h** ^**−1**^ **)** ^**d**^	**Max.** **P**_**LA**_ ** (g L** ^**−1**^ ** h** ^**−1**^ **)** ^**e**^
25	1.01±0.002	18.7±0.3	16.8±0.1^a^	0.89	0.57^a^	2.6
30	1.02±0.003	18.6±0.1	16.9±0.1^a^	0.91	0.57^a^	2.8
37	1.04±0.004	18.9±0.5	17.1±0.3^a^	0.91	0.57^a^	2.9
40	1.05±0.003	19.7±0.0	18.4±0.0^a^	0.93	0.61^a^	3.1
45	1.05±0.004	19.6±0.1	18.2±0.1^a^	0.93	0.61^a^	3.0
50	1.04±0.006	19.4±0.2	17.9±0.2^a^	0.92	0.60^a^	2.8
55	1.01±0.006	19.5±0.2	17.8±0.2^a^	0.91	0.59^a^	2.4
60	0.97±0.008	15.2±0.3	13.2±0.3^b^	0.87	0.44^b^	1.3

^a^OD, maximum optical density. ^b^Maximum lactic acid concentration after 30 h. ^c^Lactic acid yield. ^d^Lactic acid productivity at the end of fermentation time. ^e^Maximum lactic acid productivity at indicated time. Different letters between columns denote that mean values are significantly different (p≤0.05) by Tukey test; values are means ± SE (n=3).

**Table 3:** Effect of different glucose concentrations on growth and kinetic parameters of lactic acid production by *Enterococcus hirae *BoM1-2.

**Conc. of glucose (g L** ^**−1**^ **)**	**O** **D**_600 **nm**_^**a**^	**LA conc. (g L** ^**−1**^ **)** ^**b**^	**Y**_**LA**_ ** (g g** ^**−1**^ **)** ^**c**^	**P**_**LA**_ ** (g L** ^**−1**^ ** h** ^**−1**^ **)** ^**d**^	**Residual sugar (g L** ^**−1**^ **)**	**Fermentation time (h)**	**Max ** **P**_**LA**_ ** (g L** ^**−1**^ ** h** ^**−1**^ **)** ^**e**^
20	1.99±0.009	19.6±0.1^f^	0.98	2.18^a^	0.1±0.1	9	3.2 (3h)
40	4.19±0.021	37.3±0.5^e^	0.93	2.07^a^	0.0±0.0	18	3.3 (3h)
60	6.25±0.120	57.9±0.1^d^	0.97	1.75^b^	0.0±0.0	33	3.4 (3h)
80	7.69±0.074	77.4±0.0^c^	0.97	1.23^c^	0.5±0.1	63	3.1 (3h)
100	9.19±0.160	96.0±0.1^b^	0.96	1.07^d^	0.0±0.0	90	3.3 (3h)
150	9.72±0.064	109.9±0.4^a^	0.96	1.02^d^	35.7±0.4	108	2.6 (3h)

^a^OD, maximum optical density. ^b^Maximum lactic acid concentration. ^c^Lactic acid yield. ^d^Lactic acid productivity at the end of fermentation time. ^e^Maximum lactic acid productivity at indicated time. Different letters between columns denote that mean values are significantly different (p≤0.05) by Tukey test; values are means ± SE (n=3).

**Table 4:** Lactic acid production in repeated batch fermentation by *Enterococcus hirae *BoM1-2 using initial 60 g L^−1^ of glucose for the first 10 runs and 100 g L^−1^ glucose for run 11th.

Batch Number	Initial glucose (g L^−1^)	OD_600 nm_^a^	LA conc. (g L^−1^)^b^	Fermentation time (h)	*Y*_LA_ (g g^−1^)^c^	*P*_LA_ (g L^−1^ h^−1^)^d^	Max *P*_LA_ (g L^−1^ h^−1^)^e^
1	60	3.80±0.024	59.2±0.4	30	0.99	1.97	3.5
2	60	4.70±0.560	59.0±0.3	24	0.98	2.46	3.8
3	60	6.70±0.063	59.1±0.2	22	0.99	2.69	3.9
4	60	8.40±0.038	57.8±0.2	21	0.98	2.75	3.9
5	60	10.6±0.093	58.8±0.3	20	0.98	2.94	4
6	60	12.3±0.570	58.1±0.9	18	0.97	3.23	4.2
7	60	12.5±0.102	58.8±0.5	16	0.98	3.68	4.5
8	60	12.9±0.097	58.1±0.6	14	0.98	4.15	5.4
9	60	13.3±0.109	58.1±0.4	13	0.97	4.47	5.1
10	60	13.7±0.048	58.3±0.7	13	0.97	4.48	5
11	100	15.0±0.102	97.2±0.9	33	0.97	2.95	3.9

^a^OD, maximum optical density. ^b^Maximum lactic acid concentration after 30 h. ^c^Lactic acid yield. ^d^Lactic acid productivity at the end of fermentation time. ^e^Maximum lactic acid productivity at indicated time.

**Table 5:** Lactic acid production by repeated batch fermentation coupled with gradual increase in glucose concentrations from 40 to 100 g L^−1^ by *Enterococcus hirae *BoM1-2 at pH 9.0 and 40°C.

**Batch Number**	**Initial glucose (g L** ^**−1**^ **)**	**O** **D**_600 **nm**_^**a**^	**LA conc. (g L** ^**−1**^ **)** ^**b**^	**Y**_**LA**_ ** (g g** ^**−1**^ **)** ^**c**^	**P**_**LA**_ ** (g L** ^**−1**^ ** h** ^**−1**^ **)** ^**d**^	**Fermentation time (h)**	**Max ** **P**_**LA**_ ** (g L** ^**−1**^ ** h** ^**−1**^ **)** ^**e**^
**1**	40	3.10±0.196	39.6±0.2	0.99	2.20	18	3.2
**2**	40	5.40±0.030	39.8±0.2	1.0	2.49	16	3.7
**3**	40	7.10±0.016	39.7±0.2	0.99	2.84	14	4.5
**4**	40	8.80±0.001	39.7±0.1	0.99	3.31	12	4.5
**5**	40	10.1±0.012	39.9±0.3	1.0	3.99	10	4.8
**6**	40	11.4±0.013	39.6±0.5	0.99	4.95	8	6.3
**7**	40	12.3±0.020	39.7±0.2	0.99	6.62	6	7.0
**8**	40	13.9±0.016	39.6±0.3	0.99	9.90	4	11.5
**9**	40	14.9±0.0217	39.7±0.3	0.99	13.2	3	13.9
**10**	40	16.7±0.013	39.9±0.4	1.0	19.9	2	22.7
**11**	40	17.9±0.019	39.9±0.5	1.0	26.6	1.5	28.2
**12**	40	20.0±0.114	39.7±0.2	0.99	39.7	1	47.3
**13**	40	21.2±0.013	39.9±0.2	1.0	39.9	1	49.2
**14**	60	22.3±0.576	59.7±0.2	1.0	19.9	3	24.5
**15**	60	23.4±0.032	59.4±0.4	0.99	29.7	2	34.7
**16**	60	24.7±0.418	59.8±0.3	1.0	29.9	2	36.2
**17**	80	25.5±0.016	79.5±0.3	0.99	19.9	4	22.6
**18**	80	26.8±0.131	79.4±0.5	0.99	26.5	3	29.5
**19**	80	27.4±0.521	79.6±0.3	1.0	26.5	3	31.0
**20**	100	28.1±0.070	99.1±0.3	0.99	19.8	5	23.4
**21**	100	28.9±0.340	99.2±0.5	0.99	24.8	4	27.8
**22**	100	29.6±0.253	99.2±0.3	0.99	24.8	4	28.3
**23**	100	29.7±0.079	98.8±0.5	0.99	16.5	6	21.5
**24**	100	29.8±0.435	92.0±0.4	0.99	11.5	8	14.2

^a^OD, maximum optical density. ^b^Maximum lactic acid concentration after 30 h. ^c^Lactic acid yield. ^d^Lactic acid productivity at the end of fermentation time. ^e^Maximum lactic acid productivity at indicated time.

## Data Availability

The data used to support the findings of this study are available from the corresponding author upon request.

## Supplementary Materials

Supplementary Materials
**Table S1:** morphological and biochemical characterization of isolate BoM1-2.** Table S2:** sugar fermentation pattern of isolate BoM1-2 by API 50 CHL and data for* Enterococcus hirae *described by Manero and Blanch (1999).** Fig. S1:** lactic acid production and yield by the selected isolates in MSR medium containing 20 g L^−1^ glucose at 37°C for 30 h. Symbols: ■, lactic acid concentration g L^−1^; □, lactic acid yield g g^−1^ of glucose consumed.
