# Supplementary material for: High Improvement in Lactic Acid Productivity by New Alkaliphilic Bacterium Using Repeated Batch Fermentation Integrated with Increased Substrate Concentration
Source: Biomed Res Int. 2019 Jan 17;2019:7212870. doi: 10.1155/2019/7212870 (PMC6354166; doi:10.1155/2019/7212870)
Supplement: Supplementary Materials — Table S1: morphological and biochemical characterization of isolate BoM1-2. Table S2: sugar fermentation pattern of isolate BoM1-2 by API 50 CHL and data for Enterococcus hirae described by Manero and Blanch (1999). Fig. S1: lactic acid production and yield by the selected isolates in MSR medium containing 20 g L−1 glucose at 37°C for 30 h. Symbols: ■, lactic acid concentration g L−1; □, lactic acid yield g g−1 of glucose consumed. [file 7212870.f1.docx]

**Supplementary data for Article**

**-High Improvement in Lactic Acid Productivity by New Alkaliphilic bacterium Using Repeated Batch Fermentation Integrated with Increased Substrate Concentration**

**Table S1**

**Table S1:** Morphological and biochemical characterization of isolate BoM1-2.

| Character | Result | Character | Result |
| --- | --- | --- | --- |
| Cell morphology | cocci | **Extracellular enzymes** | |
| Color | white | Urea | ̶ |
|  | convex | Citrate | ̶ |
| Gram stain | + | Pectin | ̶ |
| Catalase activity | ̶ | Starch | ̶ |
| Fermentation type | Homofermentative | Cellulose | ̶ |
| **Tolerance for temperature** | | Gelatin |  |
| 25 - 60°C | + | **Tolerance to NaCl %** | |
|  |  | 2.5 | + |
| **Growth pH** |  | 5.0 | + |
| 7.0-10.0 | + | 7.5 | + |
| 11.0 | ̶ | 10 | ̶ |

+, positive; -, negative

**Table S2**

**Table S2:** Sugar fermentation pattern of isolate BoM1-2 by API 50 CHL and data for *Enterococcus hirae* described by Manero and Blanch ([1999](https://link.springer.com/article/10.1007%2Fs00253-010-2986-4#CR26)).

| No. | Substrate | BoM1-2 | *E. hirae* | No. | Substrate | BoM1-2 | *E. hirae* |
| --- | --- | --- | --- | --- | --- | --- | --- |
| 1 | Glycerol | + | d | 26 | Salicin | + | + |
| 2 | Erythitol | - | - | 27 | D-Cellobiose | + | + |
| 3 | D-Arabinose | - | ND | 28 | D-Maltose | + | + |
| 4 | L-Arabinose | + | - | 29 | D-Lactose | + | + |
| 5 | D-Ribose | + | + | 30 | D-Melibiose | + | + |
| 6 | D-Xylose | - | - | 31 | D-Sucrose | + | + |
| 7 | L-Xylose | - | - | 32 | D-trehalose | + |  |
| 8 | D-Adonitol | - | - | 33 | Inulin | - | - |
| 9 | Metyl-βD-Xylopyranoside | - | - | 34 | D-Melezitose | - | - |
| 10 | D-Galactose | + | + | 35 | D-Raffinose | - | d |
| 11 | D-Glucose | + | + | 36 | Starch | - | (+) |
| 12 | D-Fructose | + | + | 37 | Glycogen | - | - |
| 13 | D-Mannose | + | + | 38 | Xylitol | - | - |
| 14 | L-Sorbose | - | - | 39 | Gentiobiose | + | ND |
| 15 | L-Rhamnose | - | - | 40 | D-Turanose | - | ND |
| 16 | Dulcitol | - | - | 41 | D-Lyxose | - | - |
| 17 | Inocitol | - |  | 42 | D-tagatose | - | (-) |
| 18 | D-Mannitol | + | - | 43 | D-Fucose | - | - |
| 19 | D-Sorbitol | - | - | 44 | L-Fucose | - | - |
| 20 | Metyl-αD-Mannopyranoside | - | - | 45 | D-Arabitol | - | - |
| 21 | Metyl-αD-Glucopyranoside | - | - | 46 | L-Arabitol | - | ND |
| 22 | N-Acetyl-Glucosamine | + | + | 47 | Gluconate | - | - |
| 23 | Amygdalin | - | + | 48 | 2-Ketogluconate | - | - |
| 24 | Arbutin | + | + | 49 | 5-Ketogluconate | - | - |
| 25 | Escilin ferric citrate | + | ND |  |  |  |  |

+: Positive (+), 75 to 89% are positive

-: Negative (-), 11 to 25 % are positive , d, discrepancies among reference studies, ND No data

**Fig. S1:**

**Fig. S1:** Lactic acid production and yield by the selected isolates in MSR medium containing 20 g L^−1^ glucose at 37^o^C for 30h. Symbols: ■, lactic acid concentration g L^−1^; **□**, lactic acid yield g g^−1^ of glucose consumed.
